# Refractory corticotroph adenomas

**DOI:** 10.1007/s11102-023-01308-5

**Published:** 2023-03-14

**Authors:** Amit K. S. Sumal, Dongyun Zhang, Anthony P. Heaney

**Affiliations:** 1 Department of Medicine, David Geffen School of Medicine, University of California Los Angeles, Los Angeles, CA, USA; 2 Department of Neurosurgery, David Geffen School of Medicine, University of California Los Angeles, Los Angeles, CA, USA

**Keywords:** Refractory corticotroph adenomas

## Abstract

The majority of corticotroph adenomas are benign but some are locally invasive, demonstrate high rates of recurrence, and exhibit a relatively poor response to often repeated surgical, medical, and radiation treatment. Herein, we summarize the currently known somatic and genetic mutations and other molecular factors that influence the pathogenesis of these tumors and discuss currently available therapies. Although recent molecular studies have advanced our understanding of the pathogenesis and behavior of these refractory corticotroph adenomas, these insights do not reliably guide treatment choices at present. Development of additional diagnostic tools and novel tumor-directed therapies that offer efficacious treatment choices for patients with refractory corticotroph adenomas are needed.

## Introduction

Corticotroph adenomas account for 4–8% of all pituitary tumors with the majority (85%) being benign adenomas that can be successfully offered remission with transnasal trans-sphenoidal resection by an experienced pituitary surgeon [1]. However, up to 20% of these tumors exhibit high recurrence rates and/or are refractory to conventional and often repeated surgical, medical, and radiotherapeutic approaches [2]. This group of refractory corticotroph tumors includes Crooke’s cell adenomas, silent corticotroph adenomas, and corticotroph carcinomas [2]. Molecular studies have advanced our understanding of the pathogenesis and behavior of this subset of refractory corticotroph tumors and some of these may help identify tumors requiring increased sur-veillance. Presently, however, they do not guide successful treatment choices, which remain limited.

## Molecular pathogenesis of refractory corticotroph adenomas

### Germline and somatic mutations

Although the majority of corticotroph tumors are sporadic monoclonal adenomas [3], mutations in the ubiquitin-specific protease 8 (*USP8*) gene have been identified in ~ 30–50% of corticotroph tumors with a female preponderance [4–6, Table 1]. Thus far, heterozygous single-point *USP8* mutations have exclusively occurred at hotspot sites in exon 14 in a region flanking S718 (RSYSSP) [4–6]. These *USP8* mutations interrupt its interaction with a chaperone protein 14-3-3 to increase deubiquitination of the epidermal growth factor receptor (EGFR) and enhance EGFR-induced ACTH [3–6]. Interestingly, *USP8*-mutated corticotroph tumors exhibit comparatively benign behavior, and invasive and treatment-refractory corticotroph tumors more frequently exhibit a *USP8*-WT genotype [7]. One study showed that *USP8*-WT refractory corticotroph tumors harbor *TP53* mutations, contributing to a high level of chromosome instability which may underlie, at least in part, their aggressive behavior [7].

Interestingly, a further missense mutation in the catalytic domain of another deubiquitinase, *USP48* [at Met 415 (M415I/V)], has been found by whole exome sequencing in 6 out of 22 corticotroph tumors further confirmed by targeted sequencing in 16 out of 147 corticotroph tumors [8]. The mutation results in increased *USP48* activity thereby affecting NFκB activation and regulation of POMC transcription [8]. Additionally, a gain-of-function mutation in exon 9 of *PIK3CA* (G1009E) was identified in one of six invasive corticotroph tumors, and a concurrent *HRAS* G12R mutation was noted in the same tumor [9]. In other studies, lack of *ATRX* immunolabelling due to a loss-of-function *ATRX* mutation was reported in 3 refractory corticotroph pituitary tumors and 4 corticotroph-carcinomas [10]. These *ATRX* single nucleotide variants and small indels are located across the coding region of *ATRX*, and concomitant with mutations in *TP53*, *PTEN*, *RB1*, *NF2*, and/or *CDKN2A/B* and may contribute to corticotroph tumor progression in some cases [10].

In contrast, germline mutations predisposing to corticotroph tumors are relatively rare and mutations of *DICER1*, *CDK5*, *and Abl Enzyme Substrate 1* (*CABLES1*) have thus far only been reported in refractory pediatric corticotroph tumors [11–12, Table 1].

### Role of epithelial to mesenchymal transition

Epithelial to mesenchymal transition (EMT) is a dynamic functional biological process that can occur in terminally differentiated mature adult epithelial cells in response to inflammatory, hypoxic, or other pathological stressors [13]. Furthermore, EMT can work cooperatively with epigenetic and genetic changes to drive clonal tumor outgrowth, invasion, and metastatic spread [14]. EMT has previously been reported in somatostatin receptor ligand-resistant somatotroph and in *USP8*-WT corticotroph tumors [15, 16].

We recently confirmed EMT in a series of invasive silent corticotroph tumors. Single-cell RNA sequencing demonstrated increased expression of several cytoskeletal components (*ACTB*, *PFN1*, *GSN*, and *MYL12A*) which regulate granule exocytosis and cell polarization in silent – compared to functional – corticotroph tumors. In parallel with this increase in EMT transcripts in silent corticotroph tumors, we observed loss of transcripts related to hormonal biogenesis and secretion such as granin proteins (*SCG5* and *VGF*), small GTPases, and their partners (*RAB3B*, *RHOB*, *RHEB*, *ARL5B*, and *PLD3*). These changes suggest that a common transcriptional re-programming mechanism that simultaneously impairs ACTH production and activates tumor invasion may be present in silent corticotroph tumors and potentially provides additional insight into their invasive behavior [17].

## Treatment of refractory corticotroph adenomas

The designation as a refractory corticotroph tumor is largely a retrospective diagnosis, as it is often only after such tumors have failed “standard of care” medical, surgical (often multiple), and radiation therapy that they declare themselves. A systematic review of 2,653 patients demonstrates that revision surgery can offer remission in 58% of patients [18]. Radiation therapy can also offer excellent remission rates of between 55 and 100% with conventional radiotherapy and 42–81% with stereotactic approaches [19]. In a 2020 study of 45 corticotroph adenomas, which included 10 “aggressive” corticotroph tumors, gamma knife radiosurgery achieved hormonal remission in 54% and 40% and radiologic remission in 26% and 10% of patients, respectively, at 5–10 years. Hypopituitarism was observed in 15% of both patient groups [20]. It is also notable that whereas bilateral adrenalectomy is highly effective in treating hypercortisolism, it may result in corticotroph progression with a mean prevalence of 43% at a mean interval of 5.3 years [21].

Currently, no approved medical therapies exist for refractory corticotroph tumors, although treatment with the alkylating agent temozolomide has become first-line therapy for this subset. In a European Society of Endocrinology survey of 73 aggressive corticotroph tumors and carcinomas treated with temozolomide, 6 demonstrated a complete response, 22 a partial response, 20 exhibited stable disease, and 25 manifested disease progression [22].

More recently, the immune checkpoint inhibitors, ipili-mumab, nivolumab, and pembrolizumab, have been used to treat seven patients with refractory corticotroph adenomas following which four patients exhibited a partial response, one stable disease (SD), one radiologically stable disease but clinically relevant tumor growth, and one progressive disease (PD). Notably, the one patient who exhibited PD and an additional patient that exhibited SD both had bio-chemical evidence of hypercortisolism, raising the possibility that immune checkpoint inhibitors may be less effective when hypercortisolism is present though numbers of treated patients are small [1].

Two refractory corticotroph adenomas have been treated with peptide receptor radionuclide therapy (PRRT), using a radionuclide linked to a somatostatin receptor ligand. One patient died shortly after treatment, whereas the other patient developed facial pain which prevented further therapy and this subject died thirteen months later [23].

A monoclonal antibody against vascular endothelial growth factor (VEGF), bevacizumab, has also been used to treat eight refractory corticotroph adenomas following which five exhibited radiologically stable disease (one patient was concurrently treated with pasireotide and another with temozolomide), and an additional patient treated with bevacizumab in combination with temozolomide and radiotherapy showed a complete response [23, 24]. The remaining two bevacizumab-treated patients died within four months of initiating therapy [24].

All three refractory corticotroph adenomas treated with the mTOR inhibitor, everolimus ultimately developed disease progression, although one patient exhibited transient stable disease for five months prior to disease progression [24, 25]. Lastly, one refractory corticotroph adenoma that had failed to respond to temozolomide, everolimus, and bevacizumab also failed to respond to the multi-targeted tyrosine kinase inhibitor, sunitinib [25].

## Future directions

In summary, current standard of care therapy offers disease remission or at least control in the majority of patients with corticotroph tumors. However, a small subset of corticotroph tumors exhibit a poor response to combinations of currently available medical, surgical, and radiotherapeutic approaches, exhibit high rates of recurrence, and cause significant morbidity and occasional mortality in our patients. These refractory corticotroph adenomas represent an ongoing treatment challenge and further tools are needed to identify this tumor subset earlier in the disease course and guide more efficacious treatment choices. Clearly, additional novel tumor-directed therapies are needed to change disease outcomes for this patient subgroup.

## Figures and Tables

**Table 1:** List of Somatic and Germline Mutations in Refractory Corticotroph Tumors

Gene	Site	Frequency	Ref
Somatic mutations *USP8*	Region (RSYSSP) flanking S718	30–50%	[4–7]
*USP48*	M415I/V	6 out 22 cases	[8]
*PIK3CA*	G1009E	1 out of 6 cases	[9]
*HRAS*	G12R	1 out of 6 cases	[9]
*ATRX*	Multiple	7 out of 25 cases	[10]
Germline mutations			
*DICER*	Multiple	7 out of 192 cases	[11]
*CABLES*	Multiple	2.20%	[12]
